# Polypoid endometrioma mimicking malignant transformation: a case report and systematic review

**DOI:** 10.1016/j.xfre.2026.04.006

**Published:** 2026-04-22

**Authors:** Guillaume Parpex, Louis Marcellin, Joëlle Uzan-Augui, Michel Richa, Amani Hakami, Laura Larnaudie, Amel Kime, Charles Chapron, Bruno Borghese

**Affiliations:** a Department of Gynecology, Obstetrics II, and Reproductive Medicine, Centre Hospitalier Universitaire (CHU) Cochin, Hôpital Universitaire Paris Centre (HUPC), Assistance Publique-Hôpitaux de Paris (AP-HP), Paris, France; b CNRS, Inserm, Institut Cochin, Université Paris Cité, Paris, France; c Finding Answers to Manage Endometriosis, Fédération Hospitalo-Universitaire (FHU) FRAME, Paris, France; d Centre de recherche des Cordeliers, UMR1138, Unité MEPPOT, Université Paris Cité, Paris, France; e Radiology A department, Cochin Hospital, Université Paris Cité, Assistance Publique-Hôpitaux de Paris (AP-HP), Paris, France; f Department of radiology, Centre Hospitalier Universitaire (CHU) Hotel Dieu, Paris, Hôpital Universitaire Paris Centre (HUPC), Assistance Publique-Hôpitaux de Paris (AP-HP), France; g Service de pathologie, DMU IMAGINA, Centre Hospitalier Universitaire (CHU) Cochin, Hôpital Universitaire Paris Centre (HUPC), Assistance Publique-Hôpitaux de Paris (AP-HP), Paris, France

**Keywords:** Polypoid endometrioma, ovarian endometriosis, ovarian cancer mimic, magnetic resonance imaging, fertility preservation

## Abstract

**Objective:**

To report a case of polypoid ovarian endometrioma mimicking malignant transformation, review the literature on this rare entity, and highlight its implications for surgical management and fertility preservation.

**Design:**

Case report and literature review.

**Subject:**

One reproductive-age woman with suspected malignant transformation of an ovarian endometrioma.

**Exposure:**

Laparoscopic adnexectomy.

**Main Outcome Measure:**

Histopathologic confirmation of nonmalignant disease and review of surgical management in previously reported cases.

**Results:**

A 38-year-old woman desiring fertility preservation presented with a complex ovarian mass classified as O-RADS 4 on magnetic resonance imaging. Laparoscopic adnexectomy was performed for suspected malignancy. Final histopathologic examination confirmed a benign polypoid endometrioma. Review of the literature identified 22 reported cases, all managed surgically for presumed malignancy, with hysterectomy performed upfront in ten cases despite benign histology.

**Conclusion:**

Polypoid ovarian endometrioma is a rare mimic of ovarian malignancy that may lead to extensive surgery before histologic confirmation. Awareness of this entity is essential to reduce overtreatment and to support fertility-preserving management when appropriate.

## Introduction

Polypoid endometrioma is a rare form of ovarian endometriosis that can mimic malignant transformation on imaging (1). Only a few cases of polypoid endometrioma have been reported to date (2). In reproductive-age women, polypoid endometriomas may closely resemble malignant transformation of endometriosis on imaging, making accurate preoperative diagnosis particularly challenging and clinically significant. In women with endometriosis and a desire for pregnancy, suspicion of ovarian malignancy may lead to unnecessarily aggressive surgery, potentially compromising ovarian reserve and future fertility. Recognizing benign polypoid changes within an endometrioma is, therefore, crucial to avoid overtreatment and preserve reproductive potential.

We report the case of a woman of reproductive age presenting with a polypoid lesion within an ovarian endometrioma initially suspected to represent malignant transformation. Final histopathological analysis confirmed a benign endometriotic cyst with polypoid features. To contextualize this rare presentation, we performed a systematic review of the literature on polypoid endometrioma.

## Case report

A 38-year-old nulligravid woman wishing to preserve her fertility, with no significant medical history, presented with pelvic pain and abnormal uterine bleeding. Her family history was notable for breast cancer in her maternal grandmother. Six months earlier, she underwent hysteroscopic resection of an intracavitary myoma and laparoscopic drainage with alcohol sclerotherapy of a 6-cm left ovarian endometrioma. Histopathology confirmed a benign leiomyoma, and cytologic examination of the cyst fluid was negative for malignant cells. While receiving relugolix–estradiol–norethisterone acetate, she initially experienced improvement in bleeding, but symptoms recurred a few months later with renewed pelvic pain and abnormal bleeding. The patient was referred to our gynecologic oncology center.

**Figure 1:**
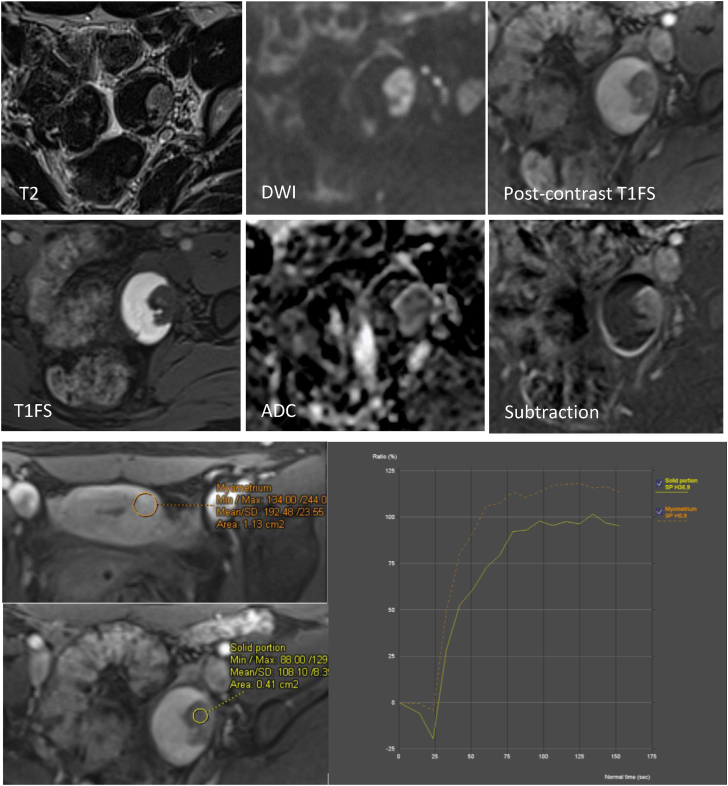
Radiologic findings of polypoid ovarian endometrioma mimicking malignant transformation. *Note:* Endometrioma with lobulated mural solid components, displaying intermediate T2 signal intensity and hyperintensity on high b-value diffusion-weighted imaging, without clearly decreased ADC values on the ADC map. Postcontrast images demonstrate enhancement of the solid portion according to a type 2 enhancement curve. ADC = apparent diffusion coefficient.

Pelvic magnetic resonance imaging (MRI) evaluation revealed a 5-cm left ovarian cystic mass with polypoid intracystic components. The lesion demonstrated heterogeneous T2 signal intensity, high signal intensity on diffusion-weighted imaging without clearly decreased apparent diffusion coefficient values (1.2 × 10^-3^ mm^2^/s), and a type-2 enhancement curve (Fig. 1). The examination was carefully reviewed by a multidisciplinary team of expert radiologists specializing in pelvic oncology and endometriosis, and the mass was classified as O-RADS 4 (3), suggesting possible malignant transformation into endometrioid or clear cell carcinoma. A 13-mm right ovarian endometrioma and several small intramural and submucosal myomas were also identified. No lymphadenopathy or peritoneal implants were detected, and tumor markers (CEA, CA-125, and CA-19-9) were within normal limits. The previously treated endometrioma was localized on the left ovary and corresponded to the lesion identified on imaging at presentation. However, given the time interval and interval changes in morphology, it was not possible to formally distinguish between recurrence of the same lesion and transformation following prior treatment.

Given the radiologic suspicion of malignancy, the case was discussed at a multidisciplinary tumor board. The right ovarian lesion, consistent with a typical endometrioma on imaging, was not surgically managed. Intraoperatively, the left ovary was enlarged by a cystic lesion. The specimen was extracted within a protective retrieval bag to avoid spillage. No peritoneal carcinomatosis was observed. A few superficial endometriotic lesions were identified in the posterior cul-de-sac. The postoperative course was uneventful.

**Figure 2:**
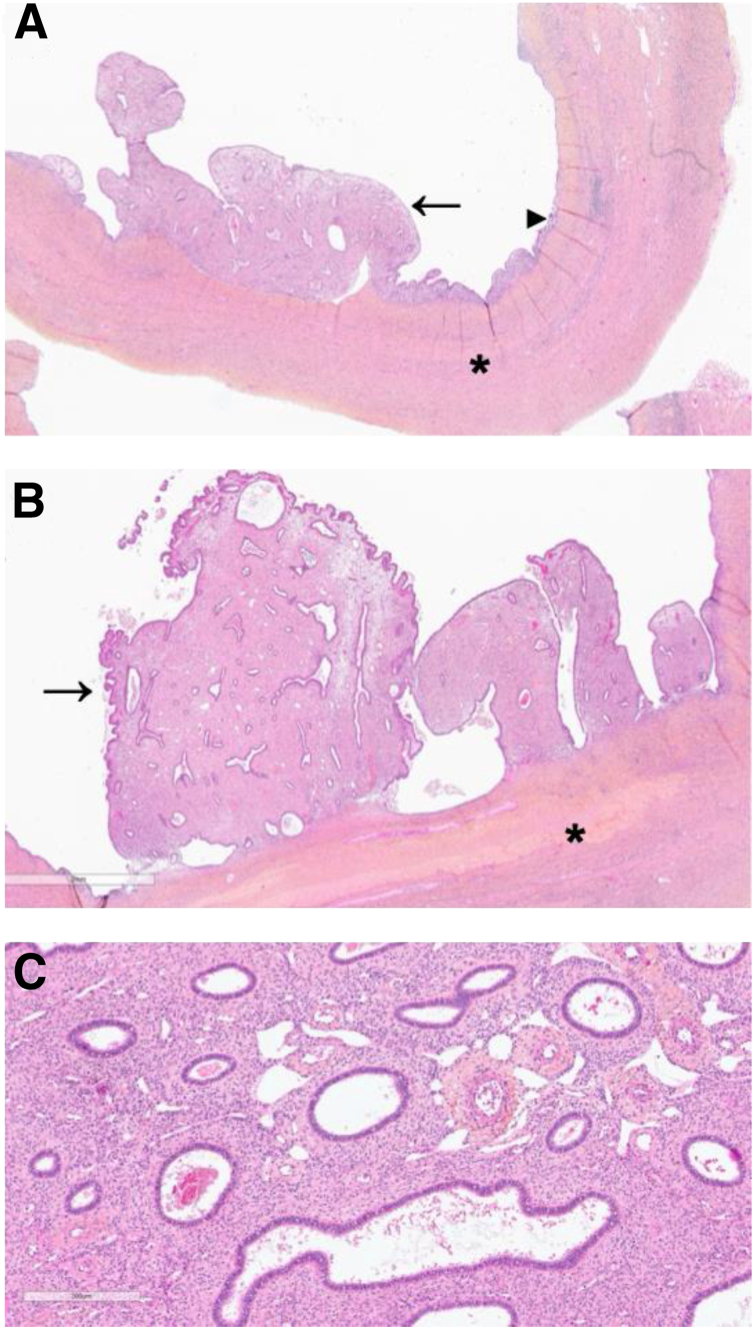
Histologic findings of polypoid endometrioma. *Note:* Hematoxylin-eosin-saffron staining. (A) and (B), Polypoid formation (arrow) arising from an endometrioma without invasion of the endometrioma wall (arrowhead) or the underlying normal ovarian tissue (asterisk). (C) Tubular glands without cytonuclear atypia irregularly scattered within a fibrous stroma containing thick-walled vessels. (Original magnifications ×1.5 [(A) and (B)] and ×10 [C]).

On macroscopic examination, the left adnexal specimen consisted of a cystic ovary measuring 5 cm, with smooth external surfaces and delicate internal membranes. The cyst contained brownish fluid and exhibited small polypoid intracystic projections without solid nodules. Microscopically, the lesion was composed of endometrial-type glands embedded in a fibrocellular stroma containing thick-walled vessels, without architectural or cytologic atypia, mitotic activity, or necrosis (Fig. 2). Residual ovarian tissue at the periphery showed small foci of endometriosis, and the fallopian tube contained limited areas of deep endometriosis without additional abnormalities. Immunohistochemical staining revealed strong and diffuse CD10 positivity in the stromal component, confirming endometrial origin. These findings were consistent with a benign polypoid endometrioma with no evidence of malignancy.

After surgery, the patient’s symptoms completely resolved. Dienogest therapy was initiated (2 mg/day), and a fertility preservation consultation was arranged in our department, considering the patient’s age and history of adnexectomy.

Written informed consent was obtained from the patient for publication of this case report and accompanying images.

## Systematic review

### Literature review methodology

A literature search was conducted in PubMed (MEDLINE), EMBASE, and Web of Science to identify articles on polypoid endometrioma published up to October 2025. Reference lists and citation sections of retrieved articles were also screened to identify additional relevant studies. The search strategy combined the following terms: (polyp OR polypoid) AND endometriosis AND (endometrioma OR ovary OR ovaries OR ovarian). This review was performed without PROSPERO registration, as it aimed to provide a descriptive synthesis of published case reports. Only studies reporting histologically confirmed ovarian polypoid endometrioma with available clinical, radiologic, or pathologic data were included. Studies were excluded if final histopathology did not confirm endometriosis or if the lesion was located outside the ovary. Full texts were assessed for eligibility according to these criteria.

### Study selection

**Figure 3:**
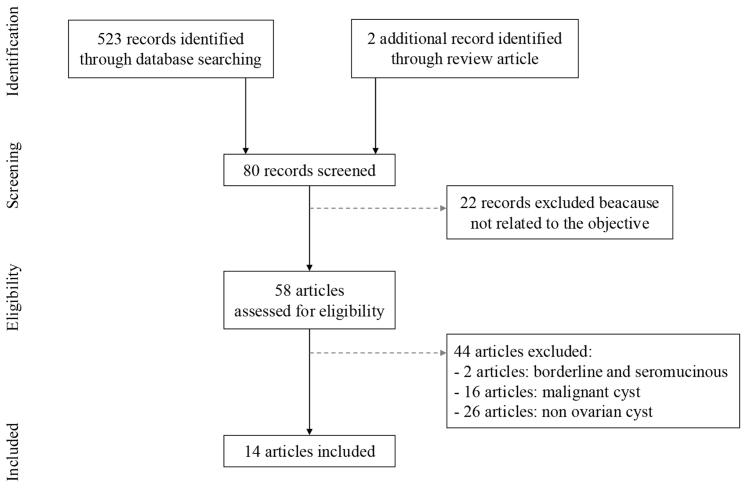
Flow chart of the study selection process.

The study selection process is presented in Figure 3. The initial search yielded 523 articles, and one additional article was identified through the review of a previously published literature review. After screening titles and abstracts, 55 articles were selected for full-text assessment. Following eligibility evaluation, 14 articles met the inclusion criteria and were retained for the final synthesis (2, 4, 5, 6, 7, 8, 9, 10, 11, 12, 13, 14, 15, 16).

## Results

A total of 14 studies reporting 22 cases of polypoid endometrioma were included, as summarized in Table 1. In the series by Parker et al. (2), which included 24 cases of polypoid endometriosis, only three cases involved the ovary and were, therefore, included in our analysis. Patients’ ages ranged from 27 to 63 years. Tumor size varied between 2 and 14 cm, and CA125 levels, when reported, ranged from normal values to 1386 U/mL. Magnetic resonance imaging most frequently showed hyperintensity on T2-weighted images, with variable T1 signal and occasional diffusion restriction or mild postcontrast enhancement. Information on associated endometriosis lesions was inconsistently reported across studies. All patients underwent surgical management with at least unilateral adnexectomy, often associated with hysterectomy (n = 10/22). Follow-up data were available in 7 of 22 cases, with two reports describing recurrence of benign polypoid endometriosis.

**Table 1:** Reported cases of polypoid endometrioma with benign histologic confirmation.

Study	Age Clinical context	Endometriosis work-up CA125^a^	MRI features of intracystic polyp	Surgery^b^	Follow-up
Anderson et al. (4) 2025 1 case	80 y Asymptomatic	Bilateral OMA 16 and 11 cm No other endometriosis lesion reported CA19-9 208 U/mL CA125 125 U/mL	Hyper T2 solid component within right OMA	Bilateral adnexectomy, hysterectomy and omental biopsy	-
Gică et al. (5) 2025 1 case	37 y Pregnant woman	OMA 6 cm No other endometriosis lesion CA125 within normal	Hyper T1 Hypo T2 DWI hyperintensity	Oophorectomy	No relapse (follow-up 6 mo)
Fan et al. (6) 2025 1 case	33 y Systemic lupus erythematosus under oral prednisolone, hydroxychloroquine, and mycophenolic acid.	OMA 12 cm Total Douglas pouch obliteration, stage IV (rASRM) CA125 1386 U/mL	-	Unilateral adnexectomy	Polypoid endometriosis relapse at 2 y (pelvic wall)
Gargan et al. (7) 2023 1 case	27 y 1 year after ipsilateral OMA excision and ovarian drilling	OMA 2 cm Stage III rASRM	Hypo T1 Homogeneous enhancement	Unilateral adnexectomy	-
Takeuchi et al. (8) 2022 2 cases	30 – 33 years	CA125 251–287 U/mL	Hyper T2 No ADC restriction Progressive enhancement DWI hyperintensity	Adnexectomy	-
Yazawa et al. (9) 2022 1 case	46 y	OMA 10 cm Stage IV rASRM CA125 113 U/mL	Iso T1 Hyper T2 DWI hyperintensity Mild enhancement T1	Unilateral adnexectomy	Polypoid endometriosis relapse at 11 months: ovary, ileum, rectosigmoid colon, omentum.
Altay et al. (10) 2021 6 cases	29 to 58 y	-	-	Adnexectomy (n=6/6), hysterectomy (n=5/6), omentectomy (n=2), pelvic lymph node dissection (n=2), nephroureterectomy (n=1)	-
Amaral et al. (11) 2021 1 case	45 y	OMA 4 cm Rectal endometriosis CA125 within normal	Hypo T2	Unilateral adnexectomy	-
Iida et al. (12) 2017 1 case	44 y	OMA 9 cm CA125 261 U/mL	Hyper T2 DWI hyperintensity	Bilateral adnexectomy, hysterectomy, omentectomy, bilateral pelvic lymph node dissection	-
Yamada et al. (13) 2014 1 case	29 y Dysmenorrhea	OMA 6 cm Superficial endometriosis CA125 122 U/mL	Hyper T2 Strong enhancement	Unilateral adnexectomy	No relapse (follow-up 28 months)
Berkes et al. (14) 2013 1 case	47 y	OMA 11 cm Polypoid tissue within OMA 2 cm No other endometriosis lesion reported CA125 484 U/mL	-	Bilateral adnexectomy, hysterectomy and omental biopsy	No relapse (follow-up 2 years)
Kozawa et al. (15) 2012 1 case	36 y	OMA 14 cm No other endometriosis lesion reported CA125 323 U/mL	Hyper T2 Hyper T1 DWI hyperintensity No ADC restriction	Unilateral adnexectomy	-
Kraft et al. (16) 2006 1 case	47 y Tamoxifen for ductal breast carcinoma	- Adenomyosis No other endometriosis lesion reported CA125 817 U/mL	Hypo T1 Hyper T2	Bilateral adnexectomy, hysterectomy and omental biopsy	No relapse (follow-up 2 months)
Parker et al. (2) 2004 3 cases^c^	63 y	OMA 4 cm No other endometriosis lesion reported	-	Bilateral adnexectomy	No relapse (follow-up 20 years)
	43 y	OMA 10 cm No other endometriosis lesion reported	-	Bilateral adnexectomy, hysterectomy and omental biopsy	-
	52 y	OMA 7 cm No other endometriosis lesion reported	-	Bilateral adnexectomy	-

Abbreviations: OMA = endometrioma, ADC = apparent diffusion coefficient, DWI: Diffusion-weighted imaging, rASRM: revised classification of American Society of Reproductive Medicine.

a Normal CA125 levels: < 35 U/mL.

b All surgical procedures were performed in a context of preoperative suspicion of ovarian malignancy.

c Of the 24 cases reported by Parker et al. (2004), only 3 involved the ovary and were included; the others were excluded because of nonovarian location.

## Discussion

The radiologic features of these benign ovarian cysts are often difficult to distinguish from malignant forms (17). This challenge is amplified by the known association between endometriosis and certain types of ovarian cancer (18, 19). The ovarian subtype of endometriosis (e.g., endometrioma) appears to carry an even higher risk of malignant transformation (20, 21). Two histologic types of ovarian cancer predominate in the context of endometriosis: clear cell carcinoma and endometrioid carcinoma (22). These aggressive cancers most often require nonconservative surgical management (23).

Such procedures, which include at minimum adnexal removal, may compromise the reproductive prognosis of affected women. This issue must also consider (i) the high prevalence of endometriosis among women of reproductive age (approximately 10%), (ii) the frequent bilaterality of endometriomas (20 to 30%), and (iii) the higher prevalence of infertility in this population, independently associated with endometriosis (24). These data support fertility preservation strategies only after histological confirmation of benignity. The literature highlights that, in several reported cases, extensive oncologic procedures—including adnexectomy and even lymphadenectomy—were performed because of suspected malignancy, whereas final histology ultimately demonstrated a benign polypoid endometrioma. This underscores that, regardless of reassuring or suspicious MRI features, definitive histopathological assessment remains mandatory, and adnexectomy is often unavoidable to establish the diagnosis. Once benignity has been confirmed, fertility preservation strategies or assisted reproductive techniques such as IVF/ICSI may be considered in patients desiring pregnancy or presenting with infertility (25).

The compilation of reported cases may help refine diagnostic criteria and support conservative management when imaging features are reassuring. In this regard, our study shows that imaging can, in some cases, revise an initially highly suspicious classification. Reassuring imaging features include the absence of solid or vascularized papillary projections, typical hemorrhagic content with T1 hyperintensity, lack of diffusion restriction, and only mild or delayed contrast enhancement. In addition, temporal comparison with previous imaging or short-interval follow-up MRI is crucial to assess lesion stability and support the benign nature of the finding. Certain other specific benign forms should also be recognized, such as tubal entrapment within an endometrioma, which can mimic a papillary projection (26). Lesion size in reported cases varied widely, ranging from 2 cm to 14 cm, but this parameter does not provide reliable information regarding the benign or malignant nature of the lesion. Conversely, this work demonstrates that CA125 levels cannot reliably differentiate between benign and malignant forms, as some cancers may present with normal markers (27), and endometriosis itself can independently cause elevated CA125 levels (28). As shown in our synthesis, CA125 levels varied widely (normal to >1,300 U/mL), confirming that this marker lacks discriminative value.

Finally, in two reported cases, a surgical procedure preceded the appearance of concerning MRI features—ovarian drilling and cystectomy (7), as well as alcohol sclerotherapy of an endometrioma in our case. These observations suggest that such interventions, even when histologic or cytologic evaluation initially confirms benignity, may be followed by the development of atypical imaging features, supporting the potential value of short-term imaging follow-up. In our case, the lesion developed in the same ovary as a previously treated endometrioma, suggesting a possible anatomical correspondence. This raises the question of a potential relationship with prior ethanol sclerotherapy. Ethanol-induced tissue injury, inflammation, and subsequent reparative processes may theoretically contribute to atypical polypoid changes. However, this hypothesis remains speculative and cannot be confirmed. In addition, the diagnostic value of cyst fluid cytology in this context remains limited, particularly for excluding malignancy in atypical lesions. Cytological analysis alone may lack sensitivity and should therefore be interpreted with caution. In cases managed by alcohol sclerotherapy, especially when imaging features are not typical, cyst wall biopsy could be considered to improve diagnostic accuracy and reduce the risk of misclassification of malignant lesions.

## Conclusion

Polypoid ovarian endometrioma is a rare but clinically significant mimic of ovarian malignancy that may lead to extensive surgery before histologic confirmation. Awareness of this entity is essential to reduce overtreatment and to guide fertility-preserving management when appropriate.

## Declaration of Interests

G.P. has nothing to disclose. L.M. has nothing to disclose. J.U-A. has nothing to disclose. M.R. has nothing to disclose. A.H. has nothing to disclose. L.L. has nothing to disclose. A.K. has nothing to disclose. C.C. has nothing to disclose. B.B. has nothing to disclose.
